# Metabolic syndrome and associated factors among *H. pylori*-infected and negative controls in Northeast Ethiopia: a comparative cross-sectional study

**DOI:** 10.3389/fendo.2024.1358411

**Published:** 2024-07-16

**Authors:** Daniel Asmelash, Marye Nigatie, Tadele Melak, Ermiyas Alemayehu, Agenagnew Ashagre, Abebaw Worede

**Affiliations:** ^1^ Department of Medical Laboratory Science, College of Medicine and Health Sciences, Mizan-Tepi University, Mizan-Aman, Ethiopia; ^2^ Department of Medical Laboratory Sciences, College of Health Sciences, Woldia University, Woldia, Ethiopia; ^3^ Department of Clinical Chemistry, School of Biomedical and Laboratory Sciences, College of Medicine and Health Sciences, University of Gondar, Gondar, Ethiopia; ^4^ Department of Medical Laboratory Sciences, College of Medicine and Health Sciences, Wollo University, Dessie, Ethiopia

**Keywords:** metabolic syndrome, *H. pylori* infection, associated factors, cross-sectional study, controls

## Abstract

**Background:**

The prevalence of metabolic syndrome (MetS) in patients infected with *Helicobacter pylori*, and the factors associated with it are not well understood. This study evaluates MetS and its associated factors among both H pylori-positive and H pylori-negative individuals in Northeast Ethiopia.

**Methods:**

A cross-sectional study was conducted between 1 March 2022 to 30 May 2022. A semi-structured questionnaire was used to collect data on sociodemographic, behavioral, and clinical variables. A total of 228 subjects were randomly selected. Blood and stool samples were collected from each subject to measure fasting blood glucose and lipid profiles, and to identify *H. pylori* infection. Data were entered into Epi. Data 3.1 and analyzed using SPSS version 25. Logistic regression analysis and the Mann–Whitney U-test were performed to determine associated factors and compare median and interquartile ranges.

**Results:**

Of the 228 participants, 114 were *H. pylori* positive, and 114 were *H. pylori* negative. Participants (50.9% female) ranged in age from 18 years to 63 years, with a median age of 31 (IQR, 22, 40) years. The overall prevalence of MetS among the participants was 23.2%. We found a statistically significant association between MetS and fasting blood glucose level (AOR, 15.965; 95% CI, 7.605–33.515, p<0.001). Furthermore, there was a statistically significant difference in the median serum levels of low-density lipoprotein cholesterol (p<0.001), triglycerides (p=0.036), systolic blood pressure (<0.001), and total cholesterol (p<0.001) between *H. pylori*-positive and *H. pylori*-negative participants.

**Conclusion:**

MetS was prevalent among study participants. There was also a statistically significant association between fasting blood sugar and MetS. In addition, systolic blood pressure, total cholesterol, triglycerides, and low-density lipoprotein levels were significantly different between *H. pylori*-positive and *H. pylori*-negative individuals.

## Introduction

Metabolic syndrome (MetS) is characterized by a cluster of metabolic abnormalities, including central obesity, impaired glucose tolerance, insulin resistance (IR), dyslipidemia, and hypertension, which markedly increase the risk of diabetes mellitus and cardio-cerebrovascular disease (1, 2). While dietary and lifestyle risk factors do not explain all the cases of MetS, other emerging risk factors are increasingly being studied (3).

*Helicobacter pylori* infection is a widespread global health issue, affecting over 50% of individuals, particularly in developing countries. The prevalence of this infection varies among different age groups and stages of development (4). *H. pylori* infection can lead to the production of inflammatory substances such as C-reactive protein (CRP), tumor necrosis factor, interferon, and interleukin-1, interleukin-6, and interleukin-8, which contribute to systemic inflammation (2, 5).

MetS affects over a quarter of the world’s population and has been linked to chronic inflammation (6). Effective management of MetS, including lowering LDL-C and raising HDL-C levels, is associated with a reduced risk of major cardiovascular events. A reduction in low-density lipoprotein cholesterol (LDL-C) by 1 mmol/L is associated with a 24% decrease in the relative risk of major vascular incidents over 5 years. Additionally, every 1 mg/dl increase in HDL-C is associated with a 2%–3% decrease in the future risk of coronary heart disease (7).

The relationship between MetS and infections can be explained by the production and secretion of pro-inflammatory cytokines, which induce metabolic manifestations (8). *H. pylori* invasion of the stomach can cause persistent stomach disturbances, which may affect some patients’ biochemical parameters (9). Potential underlying reasons for these conditions include chronic low-grade activation of the coagulation cascade, accelerated atherosclerosis, and antigenic mimicry between *H. pylori* and host epitopes, which can cause autoimmune diseases and abnormal lipid metabolism (10).

Furthermore, peptides like leptin and ghrelin, produced in the stomach, might also mediate the relationship between *H. pylori* infection and MetS (11, 12). In individuals infected with *H. pylori*, elevated levels of TC and LDL-C and reduced levels of HDL-C create an atherogenic lipid profile that may increase the risk of peripheral vascular disease, myocardial infarction, stroke, and atherosclerosis, its potential consequences (13). Specifically, the occurrence of *H. pylori* infections and reports of dyspepsia tend to rise when glycemic and metabolic control declines (14). Both the prevalence of MetS and *H. pylori* infection rise with age and are influenced by socioeconomic factors (15).

Epidemiological studies have shown mixed results regarding the association between *H. pylori* infection and MetS, with some studies indicating a significant link (16), while others do not (17, 18). This inconsistency highlights the need for further investigation, particularly in diverse populations.

Despite this, there is limited research on the impact of *H. pylori* on MetS within the Ethiopian context. Therefore, this study aims to fill this gap by investigating the prevalence of MetS and its associated factors among dyspeptic patients in northeast Ethiopia.

## Materials and methods

### Study area, design, and period

This study was conducted in the Waghimra Zone Sekota Woreda at Tefera Hailu Memorial General Hospital (THMGH), Northeast Ethiopia. A hospital-based cross-sectional study was conducted among dyspeptic patients attending THMGH from 1 March 2022 to 30 May 2022.

### Sample size and sampling technique

The sampling technique employed in this study was a stratified random sampling method designed to ensure equal representation of *H. pylori*-positive and *H. pylori*-negative dyspeptic patients attending THMGH in Northeast Ethiopia from 1 March 2022 to 30 May 2022. Initially, the study population was divided into two strata based on *H. pylori* status, and each stratum contained a list of eligible participants. Within each stratum, participants were selected using a simple random sampling technique. Specifically, a computerized random number generator was used to randomly select 114 participants from each group, ensuring a balanced and representative sample. This approach accounted for key sociodemographic characteristics, aiming to maintain balance and reduce selection bias. In cases where a selected participant was unavailable or declined participation, another individual from the same stratum was randomly chosen to replace them, thus maintaining the intended sample size and representativeness.

### Eligibility criteria

We included patients aged 18–65 who were clinically diagnosed with dyspepsia and had undergone both endoscopic examination and *H. pylori* testing. Patients with a history of chronic diseases such as diabetes mellitus, cardiovascular diseases, chronic liver, or kidney disease were excluded, as were those currently on medications that could interfere with lipid metabolism or *H. pylori* status. Additionally, we excluded patients with other gastrointestinal diseases, such as gastric cancer, to reduce heterogeneity. Subjects were screened using a two-step process: initial screening through patient medical history and clinical assessment followed by confirmatory diagnostic testing for *H. pylori* infection. Patients were excluded based on a predefined list of confounding factors. Participants who met the eligibility criteria were thoroughly informed about the study’s objectives, procedures, risks, and benefits. Written informed consent was obtained from all participants before their enrolment in the study.

### Operational definition

MetS is defined based on the modified National Cholesterol Education Program Adult Treatment Panel III Criteria (NCEP-ATP III) (19), which requires at least three of the following metabolic factors: 1) WC of ≥ 94 cm for men and ≥ 80 cm for women; 2) BP, SBP ≥ 130 mmHg or DBP ≥ 85 mmHg; 3) hyperglycemia, fast blood sugar (FBS) ≥ 100 mg/dl; 4) hypertriglyceridemia, TG ≥ 150 mg/dl; and 5) low HDL-C, HDL-C < 40 mg/dl in men or HDL-C < 50 mg/dl in women. Dyslipidemia is defined as total cholesterol (TC) >200 mg/dl and/or triglycerides >150 mg/dl and/or LDL-C > 130 mg/dl and/or HDL-C <40 mg/dl in men and/or HDL-C <50 mg/d in women (20).

Alcohol intake was defined as consuming five or more alcoholic drinks for men or four or more alcoholic drinks for women on the same occasion (i.e., at the same time or within a couple of hours) on at least 1 day in the previous month. For cigarette smoking, those who had smoked any number of cigarettes in the last 12 months were considered current smokers, while those who had stopped smoking before 12 months were considered ex-smokers. For Body Mass Index, the WHO definition of obesity is based on the body mass index (BMI) of weight-for-height: underweight (<18.5 kg/m^2^), normal weight (18.5–24.9 kg/m^2^), overweight (25.0–29.9 kg/m^2^), and obesity (≥30 kg/m^2^) (21). Hypertension was defined as systolic blood pressure ≥140 mmHg, diastolic blood pressure ≥90 mmHg, or those taking anti-hypertensive drugs (22).

### Data collection and laboratory methods

Socio-demographic characteristics and behavioral and health-related variables were collected using a structured and semi-structured questionnaire. Physical examinations, including measurements of height and weight, were performed by trained nurses to collect anthropometric data. Height (in meters) was measured using a stadiometer, and weight (in kilograms) was measured using a weighing scale in light clothing without shoes. BMI was calculated as weight in kilogram divided by height meter squared (kg/m^2^) (21).

Morning venous blood samples were collected after an overnight fast. The clotted blood samples were then centrifuged for 5 min at 3,000 rpm. The lipid panel testing utilized enzymatic colorimetric assays to measure serum glucose, total cholesterol, and triglycerides. HDL-c and LDL-c were measured using homogeneous assays, which selectively quantify HDL-c and LDL-c by selective solubilization or precipitating non-LDL-c and HDL-c and detection through spectrophotometry using a Dimension EXL Siemens clinical chemistry automation. Additionally, the stool antigen *H. pylori* Rapid Card™ InstaTest was used to diagnose *H. pylori* infection.

### Statistical analysis

The data were analyzed using SPSS version 25. Categorical variables were summarized using frequency and percentage, while continuous variables were presented as median and inter-quartile range. Mann–Whitney U-test was used for comparing continuous variables between groups using median and interquartile range. Multivariable logistic regression analysis was carried out to identify those factors statistically significant for MetS. Variables with a p-value < 0.25 in univariate analysis were included in the multivariable model (23). The Mann–Whitney U-test was used for comparing continuous variables between groups due to non-normal distributions, and logistic regression was employed for assessing associations between *H. pylori* status and MetS components.

## Results

### Sociodemographic characteristics

This study included 228 participants, with 114 *H. pylori*-positive and 114 *H. pylori*-negative individuals. Of the total, 116 (50.9%) were women, and the median (IQR) age was 31 (22, 40). The median (IQR) age of *H. pylori*-positive and *H. pylori*-negative study participants was 31 (22, 42) and 31 (20, 38) years, respectively (**Table 1**).

**Table 1:** Sociodemographic characteristics of study subjects (N=228, THMGH, Northeast Ethiopia, 2022).

Variables	Category	*H. pylori* positive N(%)	*H. pylori* negative N (%)	Total N (%)
Age (year)	18–25	41(18.0)	39(17.1)	80(35.1)
	26–35	30(13.2)	22(9.6)	52(22.8)
	36–45	36(15.8)	38(16.7)	74(32.5)
	>45	7(3.1)	15(6.6)	22(9.6)
Sex	Male	51(22.3)	61(26.6)	112(49.1
	Female	63(27.6)	53(23.3)	116(50.9)
Residence	Rural	59(51.8)	14(12.3)	73(32)
	Urban	55(48.2)	100(87.7)	155(68)
Education Status	Illiterate	39(34.2)	13(11.4)	54(23.4)
	Primary	30(26.3)	6(5.3))	36(15.5)
	Secondary	11(9.6)	55(48.2)	67(29.0)
	≥College	34(29.8)	40(35.1)	74(32.0)
Occupation	Student	25(21.9)	51(44.7)	76(33.3)
	Farmer	22(19.3)	6(5.3)	28(12.3)
	Merchant	5(4.4)	10(8.8)	15(6.6)
	Housewife	36(31.6)	15(13.2)	51(22.4)
	Gov’t employee	18(15.8)	24(21.0)	42(18.4)
	Private and others^a^	8(7.0)	8(7.0)	16(7)
Marital status	Single	31(27.2)	59(51.8)	90(39.5)
	Married	73(64)	47(41.2)	120(52.6)
	Separated^b^	10(8.8)	8(7.0)	18(7.9)

a other occupational groups.

b Separated (divorced and widowed).

### Prevalence of the MetS and components

**Figure 1:**
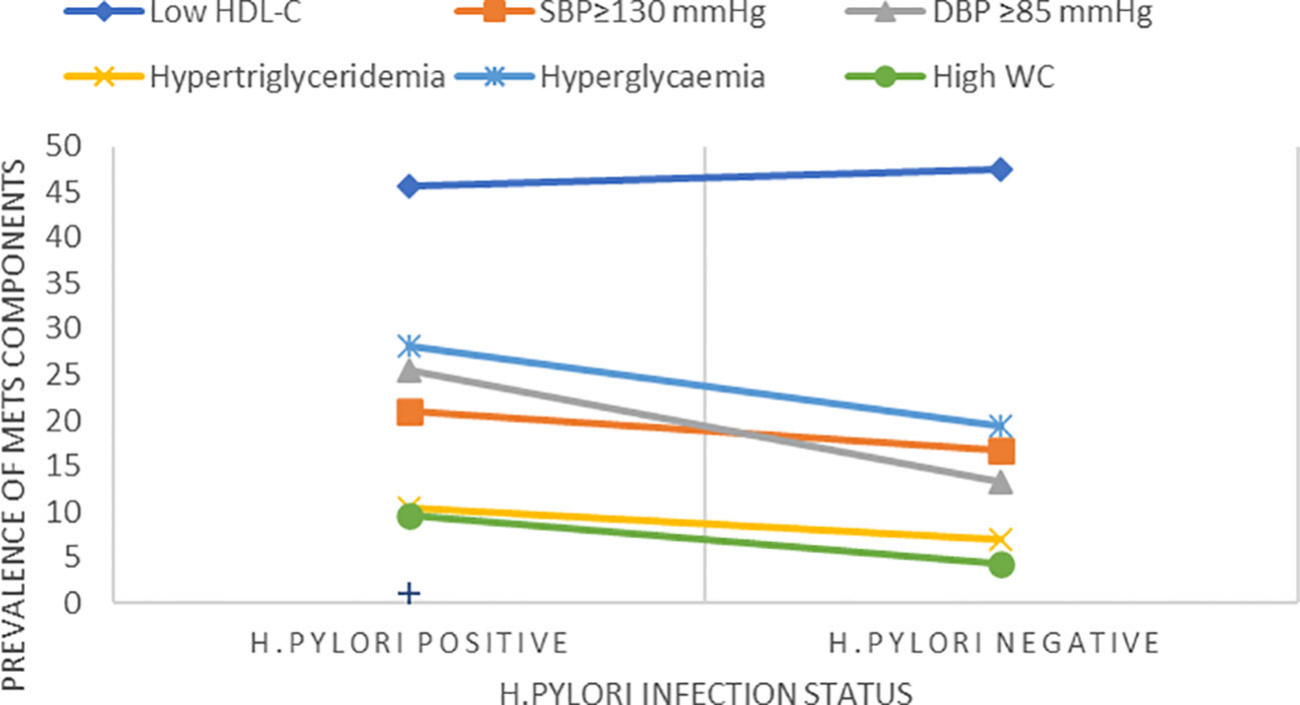
Prevalence of MetS components with *H. pylori*-positive and *H. pylori*-negative groups.

**Figure 2:**
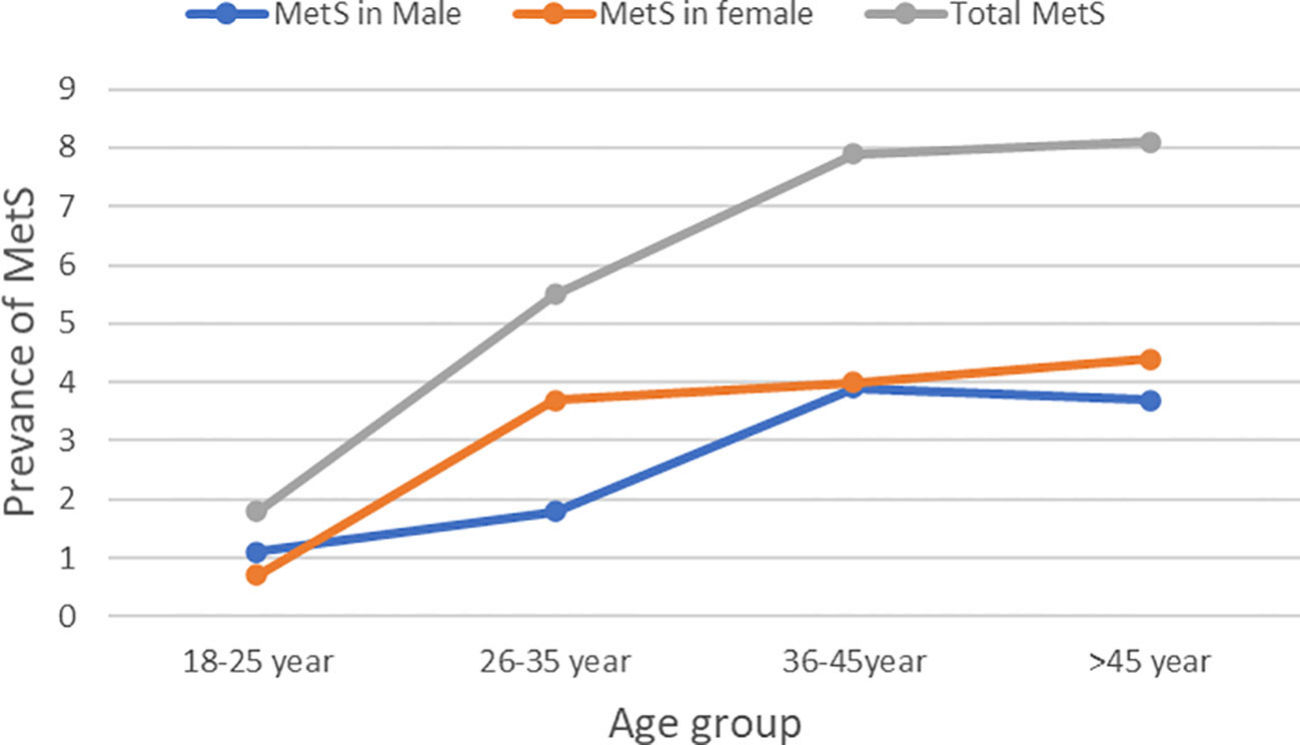
Prevalence of MetS in different age and gender.

The overall prevalence of MetS was 53(23.2%) (95% CI, 17.9%–29.3%). The prevalence of MetS was 31 (27.2%; 95% CI, 19.3%–37.3%) and 22 (19.3%, 95% CI, 12.5%–27.7%) in *H. pylori*-infected patients and *H. pylori*-negative groups, respectively (**Table 2**). Of the study participants, 54 (23.7%) were hyperglycemic. The prevalence of hyperglycemia was 32 (28.1%) and 22 (19.3%) in the *H. pylori*-infected and *H. pylori*-negative groups, respectively. Low HDL-c 106(46.5%) and hypertriglycemia 54 (23.7%) were the two most common MetS components found among all study participants (**Figure 1**, **Table 3**). However, none of the participants reported smoking. In our study, female participants had a higher prevalence than male participants throughout the age group (**Figure 2**).

**Table 2:** Associated factors of MetS among study participants Northeast Ethiopia.

Variables		MetS		COR (95% CI)	AOR (95% CI)
		Yes n	No n		
Age	18–25 years	4	76	1	1
	26–35 years	12	40	1.517(0.873–2.76)	2.708(0.779–5.188)
	66–45 years	18	56	0.930(0.610–3.078)	1.718(0.343–8.597)
	>45 years	18	4	1.401 (0.6754–2.610)	1.6779(0.692–4.063)
Sex	Male	24	88	1	1
	Female	29	87	1.222(0.660–2.265)	0.860(0.364–2.033)
Marital status	Single	14	76	1	1
	Married	32	88	1.974(0.981–3.971)	2.697(0.560–12.997)
	Separated	7	11	3.455(1.143–10.439)*	8.732(1.214–62.794)*
Residence	Rural	14	59	1	1
	Urban	39	114	1.417(0.713–2.815)	2.308(0.869–6.128)
Education status	Unable to read and write	14	38	0.930(0.420–2.058)	1.718(0.343–8.597)
	Primary	7	29	0.609(0.231–1.604)	1.182(0.258–5.421)
	Secondary	11	55	0.505(0.222–1.148)	1.342(0.380–4.731)
	College and above	21	53	1	1
Occupation status	Student	12	64	1	1
	Farmer	5	23	1.159(0.368–3.650)	0.427(0.060–3.063)
	Merchant	4	11	1.939(0.529–7.15)	0.408(0.051–3.292)
	Housewife	13	36	1.825(0.756–4.405)	0.631(0.107–3.722)
	Gov’t employee	16	26	3.282(1.366–7.884) *	0.798(0.168–3.788)
	Private and others	3	13	1.231(0.304–4.984)	0.192(0.017–2.169)
Alcohol drinking	Yes	2	14	2.217(0.488–10.085)	7.531(0.982–6.472)
	No	51	161	1	1
BMI	18.5–24.99 kg/m^2^	40	142	1	1
	>24.99 kg/m^2^	7	10	2.485 (0.889–6.944)	2.286(0.614–8.505)
	<18.5	6	23	0.926(0.353–2.430)	1.869(0.679–5.139)
Exercise	Yes	9	25	1	1
	No	44	150	1.227(0.534–2.822)	1.734(0.652–4.614)
*H. pylori* status	Negative	22	92	1	1
	Positive	31	83	1.562(0.839–2.909)	3.115(0.637–6.266)
WC	<94 cm	47	165	1	1
	≥94 cm	6	10	2.105(0.728–6.096)	1.354(0.126–14.533)
Hyperglycemia	<100 mg/dl	18	156	1	1
	≥100 mg/dl	35	19	14.56(7.204.–28.150) *	15.965(7.605–33.515)*

*p<0.05.

CI, confidence interval; AOR, adjusted odds ratio; BMI, body mass index; COR, crude odds ratio; HC, hip circumference; MetS, metabolic syndrome; WC, waist circumference.

**Table 3:** MetS among *H. pylori*-positive and *H. pylori*-negative patients (N=228 at THMGH, Northeast Ethiopia, 2022).

Variables	*H. pylori* status				Total	
	Positive		Negative			
	N (%)	95% CI	N (%)	95% CI	N (%)	95% CI
Low HDL-C	52(45.6)	36.3–55.2	54(47.4)	37.9–56.9	106(46.5)	39.9–53.2
SBP ≥ 130 mmHg	24(21.1)	14–29.7	19(16.7)	10.3–24.8	43(18.9)	14–24.6
DBP ≥ 85 mmHg	29(25.4)	17.7–34.4	15(13.2	7.6–20.8	44(19.3)	14.4–25.0
Hypertriglyceridemia	12(10.5)	5.6–11.7	8(7.0)	3.1–13.4	20(8.8)	5.4–13.2
Hyperglycemia	32(28.1)	20.1–37.3	22(19.3)	12.5–27.5	54(23.7)	18.3–29.7
WC ≥ 94 cm for men or ≥80 cm for women	11(9.6)	4.9–16.6	5(4.4)	1.4–9.9)	16(7.0)	4.1–11.1
MetS	31(27.2)	19.3–37.3	22(19.3)	12.5–27.7	53(23.2)	17.9–29.3

CI, confidence interval; HDL, high-density lipoprotein; LDL, low-density lipoprotein; MetS, metabolic syndrome.

### Factors associated with MetS

In bivariate analysis, marital status, education status, occupation, and hyperglycemia were found to have statistically significant associations with MetS (p < 0.05). However, in the multivariable analysis, only fasting blood glucose (FPG) and marital status showed a statistically significant association with MetS. In addition, our analysis did not find a statistically significant association between *H. pylori* status and the frequency of metabolic syndrome (**Table 2**).

### Biochemical and anthropometric comparisons

**Figure 3:**
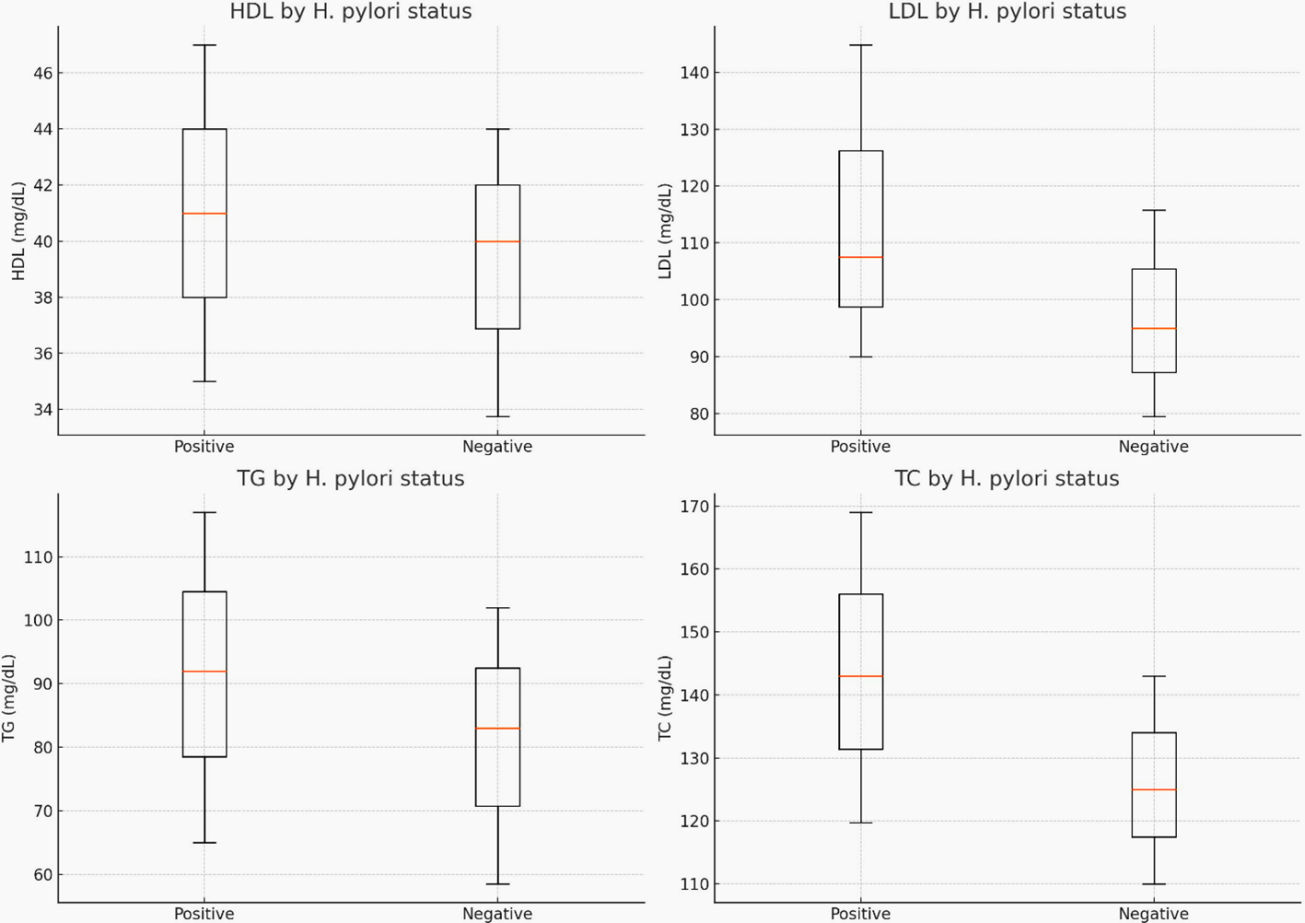
The median (IQR) for cholesterol, triglyceride, LDL-C, and HDL-C levels by *H. pylori*-infection status.

Participants with *H. pylori* infection had significantly higher median serum LDL-C (p < 0.001), TG (p = 0.036), TC (p < 0.001), and SBP (p <0.001) compared to *H. pylori*-negative participants (**Table 4**) and (**Figure 3**).

**Table 4:** Comparison of biochemical and anthropometric variables among *H. pylori*-positive and *H. pylori*-negative study participants (Mann–Whitney U test).

Variables	*H. pylori status*		p-value
	Positive, median (IQR)	Negative, median (IQR)	
Age	31(22,42)	31(20,38)	0.076
WC	75(69,86.25)	77(70.75,82)	0.811
SBP	110(105,115)	110(110,120)	<0.001
DBP	70(65,80)	70(70,80)	0.086
FBS	90(83.75,100)	90(84,97.25)	0.544
HDL	41(35,47)	40(33.75,44)	0.361
LDL	107.5(90,144.85)	95(79.45,115.8)	<0.001
TG	92(65,117)	83(58.5,102)	0.036
TC	143(119.75, 169)	125(110,143)	<0.001

DBP, diastolic blood pressure; FBS, fast blood sugar; HDL, high-density lipoprotein; HC, hip circumference; LDL, low-density lipoprotein; SBP, systolic blood pressure; TC, total cholesterol; TG, triglycerides; WC, waist circumference; p<0.05 = statistically significant value.

## Discussion

The total prevalence of MetS among study participants was 23.2% (95% CI, 17.9–29.3), which was higher than a study conducted in Taiwan in 2015 (6.5%) (24). However, our findings were lower than those of a study conducted in the USA (47.8%) (25), in Lebanon in 2020 (37%) (17), and in China in 2016 (44.5%) (26). The observed variation in prevalence can be attributed to difference in the underlying important risk factors such as urbanization, westernization, lifestyle including unhealthy diet and physical inactivity, mental stress due to economic, social, and cultural factors (27, 28). The impact of intrauterine growth retardation, overnutrition, and a high prevalence of undernutrition further complicates this picture, reflecting the multifaceted nature of MetS risk factors (29).

The magnitude of MetS among *H. pylori*-positive study individuals was 27.2% (95% CI, 19.3–37.3), which aligns with a similar prevalence reported in Korea (25). However, the prevalence was lower than in Lebanon (46.5%) (17) and China (53.7%) (26) but higher than a study conducted in Taiwan (12.4%) (24). Among *H. pylori-*negative patients, the prevalence was 19.3% (95% CI, 12.5–27.7%), consistent with a Korean study that reported a prevalence of 21% (25) but lower than Chinese studies (44.8%) (30) and (53.7%) (26). These differences can be ascribed to lifestyle, population characteristics, study designs, eligibility criteria, and sample sizes.

In the current study, the odds of MetS among people with FPG levels ≥100 mg/dl were 15.965 times higher (AOR, 15.965; 95% CI, 7.605–33.515, p<0.001) than in those with FPG levels <100 mg/dl. This finding was consistent with studies in Jimma, Ethiopia (31) and Korea (25), suggesting that hyperglycemia, driven by insulin resistance, is a critical component of MetS. Insulin resistance impairs glucose uptake in peripheral tissues and increases hepatic glucose production, underscoring the importance of glycemic control in managing MetS risk. However, our analysis did not find a statistically significant association between *H. pylori* status and the frequency of metabolic syndrome. This suggests that other factors may play a more critical role in the development of metabolic syndrome.

The study reports a statistically significant difference in LDL-C and TC levels between *H. pylori*-positive and *H. pylori*-negative individuals, with no statistically significant difference in HDL-C levels. This may be due to the effect of H. pylori infection on lipid metabolism (32).

The higher median levels of LDL-C, TG, TC, and SBP among *H. pylori*-positive individuals compared to *H. pylori*-negative ones were consistent with findings from studies conducted in Iraq (33) and Japan (34). Additionally, a Turkish study found that *H. pylori*-infected patients had significantly higher TC levels compared to *H. pylori*-negative individuals. Furthermore, the *H. pylori*-positive group exhibited higher serum TG levels than H. pylori-negative individuals (35).

In addition, the current findings were supported by those of a study conducted in Sudan (36), China (37), Korea (38), and Korea (25), which found a statistical difference between patients and controls in TC, TG, and LDL-C values. This difference is believed to be due to a disturbance in food absorption in the digestive system, leading to changes in serum lipids. The impact of *H. pylori* infection on the inflammatory response system may contribute to these alterations in lipid profiles (39). Gram-negative bacteria like *H. pylori* contain LPS that triggers the release of high levels of cytokines (TNF-a and IL-6). These cytokines inhibit the action of lipoprotein lipase, leading to the mobilization of fat from tissues into the bloodstream and subsequently increasing serum lipid levels (40, 41).

However, these findings contradicted a study conducted in Finland (42), which found no significant difference in serum TC, LDL-C, and TG levels between *H. pylori*-positive and *H. pylori*-negative individuals, although a significant difference was observed in HDL-C levels. This discrepancy may be due to population-specific factors, different methodologies, or variations in the duration and severity of *H. pylori* infection.

The limitations of the study include the lack of controls for participants’ familiarity with metabolic syndrome and *H. pylori* infection, the exclusion of genetic factors, and the oversight in accounting for dietary habits, integral to metabolic health, may have confounded our results. Addressing these limitations in future studies is essential to offer a more holistic understanding of the relationship between *H. pylori* infection and metabolic syndrome.

## Conclusion

This study found a high prevalence of MetS among dyspeptic patients, and hyperglycemia was a significant associated factor. SBP, LDL-c, TG, and TC showed a significant difference between *H. pylori*-positive and *H. pylori*-negative individuals. These findings suggest that *H. pylori* infection causes lipid metabolism disorders that increase the risk of cardiovascular diseases. This emphasizes the need for healthcare providers to consider *H. pylori* status when assessing cardiovascular risk and to incorporate lipid profile monitoring into the management plan for these patients.

## Data availability statement

The original contributions presented in the study are included in the article/supplementary material. Further inquiries can be directed to the corresponding author.

## Ethics statement

The studies involving humans were approved by the Ethical review committee of the College of Health Sciences, Woldia University with reference number WU/CHSEC/1235/22. The studies were conducted in accordance with the local legislation and institutional requirements. The participants provided their written informed consent to participate in this study.

## Author contributions

DA: Conceptualization, Data curation, Formal analysis, Funding acquisition, Investigation, Methodology, Project administration, Resources, Software, Supervision, Validation, Visualization, Writing – original draft, Writing – review & editing. MN: Conceptualization, Data curation, Formal analysis, Funding acquisition, Investigation, Methodology, Project administration, Resources, Software, Supervision, Validation, Visualization, Writing – original draft, Writing – review & editing. TM: Data curation, Investigation, Methodology, Project administration, Resources, Validation, Visualization, Writing – original draft, Writing – review & editing. EA: Conceptualization, Investigation, Methodology, Project administration, Resources, Software, Supervision, Validation, Writing – original draft, Writing – review & editing. AA: Conceptualization, Data curation, Investigation, Software, Supervision, Validation, Visualization, Writing – original draft, Writing – review & editing. AW: Conceptualization, Data curation, Formal analysis, Funding acquisition, Investigation, Resources, Software, Validation, Visualization, Writing – original draft, Writing – review & editing.
